# Embryo with XYY syndrome presenting with clubfoot: a case report

**DOI:** 10.4076/1757-1626-2-8404

**Published:** 2009-09-01

**Authors:** Dimitrios Athanatos, Christos Tsakalidis, George P Tampakoudis, Maria N Papastergiou, Fillipos Tzevelekis, George Pados, Efstratios A Assimakopoulos

**Affiliations:** 1 First Department of Obstetrics and Gynaecology, Aristotle University of Thessaloniki, ‘Papageorgiou’ HospitalThessalonikiGreece; 2 Second Department of Neonatal Intensive Care Unit, Aristotle University of Thessaloniki, ‘Papageorgiou’ HospitalThessalonikiGreece

## Abstract

Talipes equinovarus (clubfoot) is a skeletal anomaly of the embryo’s legs, with a frequency of 1-3:1000 living born babies. It may occur as an independent anomaly, or as part of a syndrome with concomitant chromosomal abnormalities.

XYY syndrome is a quite rare sex chromosomal abnormality with 47, XYY karyotype. Prenatal diagnosis is usually accidental because the syndrome is not associated with increased prevalence of sonographically detectable defects. The possibility of co-existence of skeletal anomalies in embryos with 47, XYY karyotype is scant, with only a few cases reported in the literature.

An amniocentesis was performed in an embryo at the 21^st^ week of gestation because clubfoot was detected in the 2^nd^ trimester scan, and the embryo was found to have abnormal karyotype of 47, XYY. Current opinions and management dilemmas are discussed.

## Introduction

Talipes equinovarus (clubfoot) is a skeletal anomaly of the fetal legs occurring every 1-3:1000 live births [1]. This malformation may be found as a single anomaly (idiopathic clubfoot), or in conjunction with other chromosomal and neuromuscular abnormalities.

Karyotype XYY is a rather rare chromosomal abnormality occurring in 1:1000 live births. Prenatal diagnosis is usually accidental since the syndrome is not associated with increased prevalence of sonographically detectable defects. Most of the cases diagnosed prenatally involve pregnant women undertaking amniocentesis for variable reasons (parents wish, maternal age, abnormal biochemical or sonographic markers). Individuals with 47, XYY karyotype do not share severe handicaps or specific characteristics, so mostly remain undiagnosed through their lives.

We present a case of an embryo with clubfoot found to have abnormal karyotype of 47, XYY. Current opinions and management dilemmas are discussed, as well as parents final decision is reported.

## Case presentation

**Figure 1.**
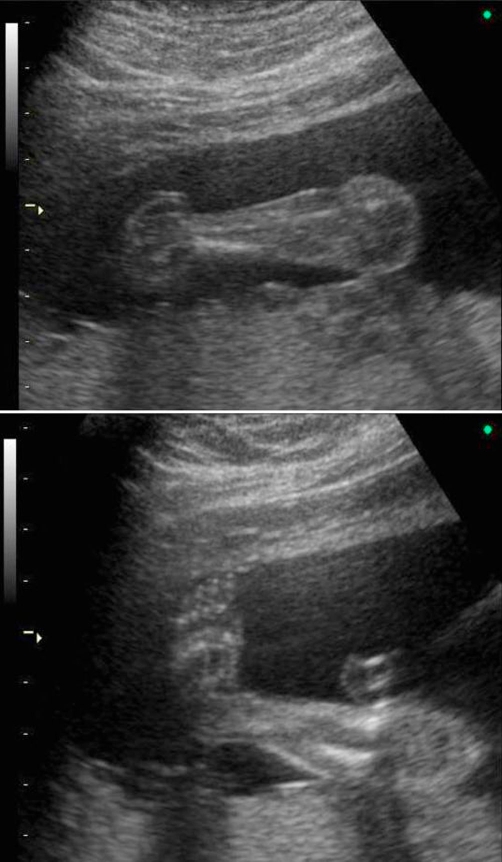
Ultrasound scan showing talipes equinovarus.

A 29-years old Caucasian woman (gravida1, para 0) referred to our department on the 23rd week of her pregnancy for a medical termination of pregnancy, due to diagnose of embryonic abnormal karyotype of 47, XYY. She had been subjected to amniocentesis 2 weeks earlier after her 2nd trimester scan (anomaly scan), because bilateral clubfoot was found (Figure 1) as a single anatomical defect.

**Figure 2.**
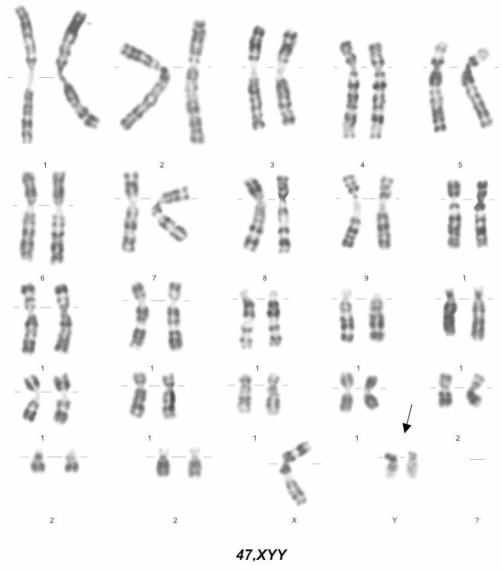
Amniocentesis results showing 47, XYY karyotype.

Proper counseling was offered in both parents after the 2nd trimester scan regarding the skeletal anomaly that was found and the need or not of an amniocentesis in order to exclude a chromosomal anomaly. The couple decided to have an amniocentesis and an abnormal karyotype of 47, XYY was detected (Figure 2). Following the diagnosis up-to-date counseling was offered in the parents regarding 47, XYY syndrome and the couple decided lawful to terminate their pregnancy immediately.

The lady was admitted in our department and both parents gave full consent for the termination of the pregnancy. Medical abortion was commenced with misoprostol, 800 micrograms at the beginning and 200 micrograms every four hours. A total dose of 1400 micrograms of misoprostol was needed for the full expulsion of the embryo and the placenta, while the integrity of the endometrial cavity was checked after the abortion by performing an ultrasound scan. The embryo weighted 580 gr. and the pathological report confirmed the sonographically findings. The lady remained in the ward for 24 hours and then she was discharged home.

## Discussion

Talipes equinovarus (clubfoot) is a skeletal anomaly of the fetal legs occurring every 1-3:1000 live births [1]. It can be idiopathic without any other anomaly, or part of syndrome with neuromuscular and chromosomal anomalies. Especially in cases of non-idiopathic clubfoot there are concomitant neuromuscular abnormalities in up to 54% of them [2]. The severity of the abnormality is not always the same and the therapy provided by orthopaedics is individualized. Nowadays early detection of clubfoot is achieved in-utero by ultrasound scan in the second trimester of pregnancy, offering valuable information to the couple about their child’s wellbeing. The questions that arise every time a sonographer detects this skeletal anomaly is whether it is idiopathic or part of a syndrome with chromosomal abnormalities, as well as if amniocentesis is necessary or the specialized sonographer can be reassuring [3,4].

Genetic involvement in the pathogenesis of talipes equinovarus reported for the first time in 2005 by Heck et al. [5], who showed that micro-deletions in chromosome 2q31-33 was a constant finding in families with idiopathic clubfoot, while more recent papers confirmed the genetic background of this skeletal anomaly [6].

Reviewing the literature on whether amniocentesis should be offered when clubfoot is diagnosed during 2nd trimester scan did not reveal any definite guidelines. According to Canto et al. [7], up to 7.1% of cases with idiopathic clubfoot appear to have some chromosomal abnormality (surprisingly not a specific one) and amniocentesis should always be offered in these couples, while other authors find a much weaker correlation and do not propose an amniocentesis [8-10].

Karyotype 47, XYY is a rare chromosomal abnormality occurring in 1:1000 live births. The phenotype of these individuals is in general normal, so prenatal diagnosis of the syndrome is rather accidental.

Men with 47, XYY karyotype do not share certain or common characteristics, making the diagnosis by the phenotype quite risky. Individuals with 47, XYY are often short with low Body Mass Index (BMI) [11], present various malformations of their urogenital tract [12], and their IQ is generally 10-15 points below average [13,14]. Although hostility was considered in the past quite common among them, it seems that they do not differ from the general population.

Correlation between idiopathic clubfoot and karyotype 47, XYY has only been found in two cases in the international literature, so it seems that this karyotype does not cause the certain skeletal anomaly and this is rather accidental [7,15].

In our case clubfoot was an isolated anomaly, proper counseling was offered and the couple decided to undertake the amniocentesis. Following the karyotype results extensive counseling regarding 47, XYY syndrome was given and both parents decided to have a lawful medical abortion, mainly due to the fact that this syndrome, although not fatal, may cause certain problems to the individual.

## Conclusions

Clubfoot in embryos with karyotype 47, XYY is a rather rare combination. Amniocentesis in cases with idiopathic clubfoot is definitely a management dilemma. Proper and up-to-date counseling should always be offered in cases with idiopathic clubfoot and the couple should take final decision upon amniocentesis freely, since no one can be definitely reassuring.
